# Brain Magnetic Resonance Imaging in Wilson’s Disease—Significance and Practical Aspects—A Narrative Review

**DOI:** 10.3390/brainsci14070727

**Published:** 2024-07-19

**Authors:** Tomasz Litwin, Barbara Rędzia-Ogrodnik, Agnieszka Antos, Adam Przybyłkowski, Anna Członkowska, Jan Paweł Bembenek

**Affiliations:** 1 Second Department of Neurology, Institute of Psychiatry and Neurology, 02-957 Warsaw, Poland; kasiaredzia@poczta.onet.pl (B.R.-O.); agantos@ipin.edu.pl (A.A.); czlonkow@ipin.edu.pl (A.C.); 2 Department of Gastroenterology, Medical University, Warsaw 02-097, Poland; aprzybylkowski@interia.pl; 3 Department of Neurophysiology, Institute Psychiatry and Neurology, 02-957 Warsaw, Poland; jbembenek@ipin.edu.pl

**Keywords:** Wilson’s disease, copper, magnetic resonance imaging, brain

## Abstract

Wilson’s disease (WD) is a genetic disorder of copper metabolism with pathological copper accumulation in many organs, resulting in clinical symptoms, mostly hepatic and neuropsychiatric. As copper accumulates in the brain during WD, and almost 50% of WD patients at diagnosis present with neurological symptoms, neuroimaging studies (especially brain magnetic resonance imaging (MRI)) are part of WD diagnosis. The classical sequences (T1, T2, and fluid-attenuated inversion recovery) were used to describe brain MRI; however, with the development of neuroradiology, several papers proposed the use of new MRI sequences and techniques like susceptibility-weighted images, T2*, diffusion MRI, tractography, volumetric assessment and post-processing brain MRI analysis of paramagnetic accumulation—quantitative susceptibility mapping. Based on these neuroradiological data in WD, currently, brain MRI semiquantitative scale and the pathognomonic neuroradiological brain MRI signs in WD were proposed. Further, the volumetric studies and brain iron accumulation MRI analysis suggested brain atrophy and iron accumulation as biomarkers of neurological WD disease severity. All these results highlight the significance of brain MRI examinations in WD. Due to the extreme progress of these studies, based on the available literature, the authors present the current state of knowledge about the significance, practical aspects, and future directions of brain MRI in WD.

## 1. Introduction

Wilson’s disease (WD) is a genetic disorder of copper metabolism caused by a defect in ATPase7B, which is highly expressed in the liver and brain. This enzyme is involved in copper transport, specifically in the export of copper from hepatocytes to bile and its incorporation into ceruloplasmin (Cp) [1,2,3]. In healthy conditions, copper bound to ceruloplasmin (Cp) is released into the systemic circulation and transported to various tissues. In the course of WD, the absence of this protein/enzyme leads to initial copper accumulation in hepatocytes, resulting in their damage (necrosis, cuproptosis, etc.), producing clinical symptoms of liver injury. Subsequently, the release of non-ceruloplasmin-bound copper (NCC), also known as ‘free/toxic copper’, into the blood leads to copper accumulation in other tissues, causing clinical symptoms primarily related to neuropsychiatric processes [4,5,6,7,8,9,10,11]. As ATPase7B has the highest expression in the liver and brain, copper accumulates in all organs; however, its concentration is highest in the brain and liver, leading to hepatic and neuropsychiatric symptoms, which was documented by Wilson [2].

The initial radiological studies, performed in the early 1980s in WD patients using computed tomography (CT), documented mainly brain atrophy (cortex, posterior fossa, brainstem, ventricular dilatation), especially in WD patients with neurological symptoms. In the most severe cases, hypointensity in basal ganglia was visualized; however, this assessment had limited significance in WD management [2,9]. It was mostly useful for excluding tumors, hematomas, or other organic lesions of the central nervous system as a cause of neurological symptoms. The advent of brain magnetic resonance imaging (MRI) has significantly advanced our understanding of WD. Clear visualization of typical WD lesions in the basal ganglia, whether reversible or irreversible, is incorporated into the WD diagnostic algorithm (Leipzig score). The identification of pathognomonic neuroradiological signs on brain MRI, the development of a brain MRI semiquantitative scale for WD monitoring, and volumetric studies highlighting the significance of atrophy, particularly in neurological patients, have all contributed to these advancements. Additionally, the documentation of brain MRI changes, even in WD patients without neurological symptoms, has fundamentally altered our knowledge of the disease, enhancing diagnostic capabilities and improving treatment monitoring [10]. Currently, many new brain MRI techniques and post-processing software (such as volumetric FreeSurfer and SIENAX 2.6/FSL 6.0), as well as methods for assessing brain iron accumulation (quantitative susceptibility mapping, QSM), are being developed and proposed to verify our knowledge and enhance treatment possibilities in WD [9,10,11]. Thus, the authors conducted a narrative review to document the significance and practical aspects of brain MRI examinations in WD, as well as to outline future directions in the neuroradiological assessment of WD patients.

## 2. Materials and Methods

We conducted a targeted literature review of articles available in the PubMed database, limited to those published in English. The search terms included ‘Wilson’s disease/Wilson disease’, ‘magnetic resonance imaging’, ‘MRI’, ‘diffusion’, ‘susceptibility-weighted imaging’, and ‘QSM’, covering publications from inception to 27 June 2024. Subsequently, we evaluated the articles and abstracts based on their significance and relevance to our study. Below, we present a summary of the data collected in the form of a narrative review.

## 3. Brain MRI in WD—Sequences and Its Significance

### 3.1. Classical Brain MRI Examination in WD Patients

**Figure 1:**
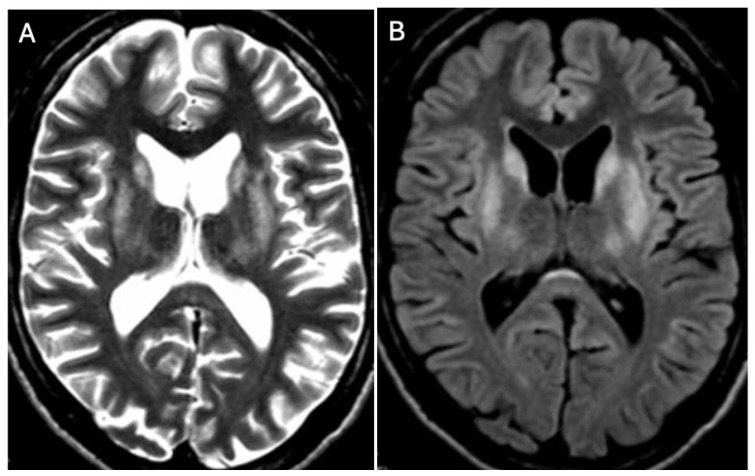
Hyperintense changes localized in putamen, head of nucleus caudate, and thalamus in T2-weighted sequences (**A**) and FLAIR (**B**) (own materials of the neurology department).

**Figure 2:**
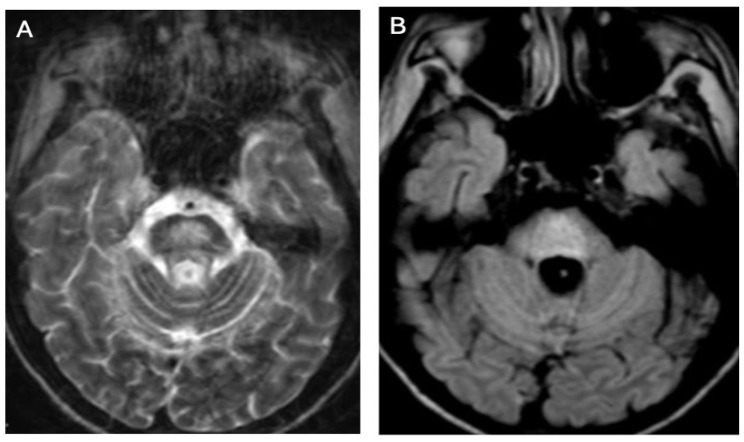
Hyperintense changes localized in pons in T2-weighted images (**A**) and FLAIR (**B**) (own materials of the neurology department).

Brain MRI appears to be the most valuable neuroradiological examination for diagnosing WD, differentiating extrapyramidal symptoms, and monitoring anti-copper treatment. Classical brain MRI findings in WD typically include symmetric (rarely asymmetric) hyperintense or mixed-intensity changes in the basal ganglia (globus pallidus, caudate nucleus, thalamus, and/or pons) observed in fluid-attenuated inversion recovery (FLAIR) and T2-weighted sequences [9,12,13,14,15,16,17,18] (Figure 1A,B and Figure 2A,B).

**Figure 3:**
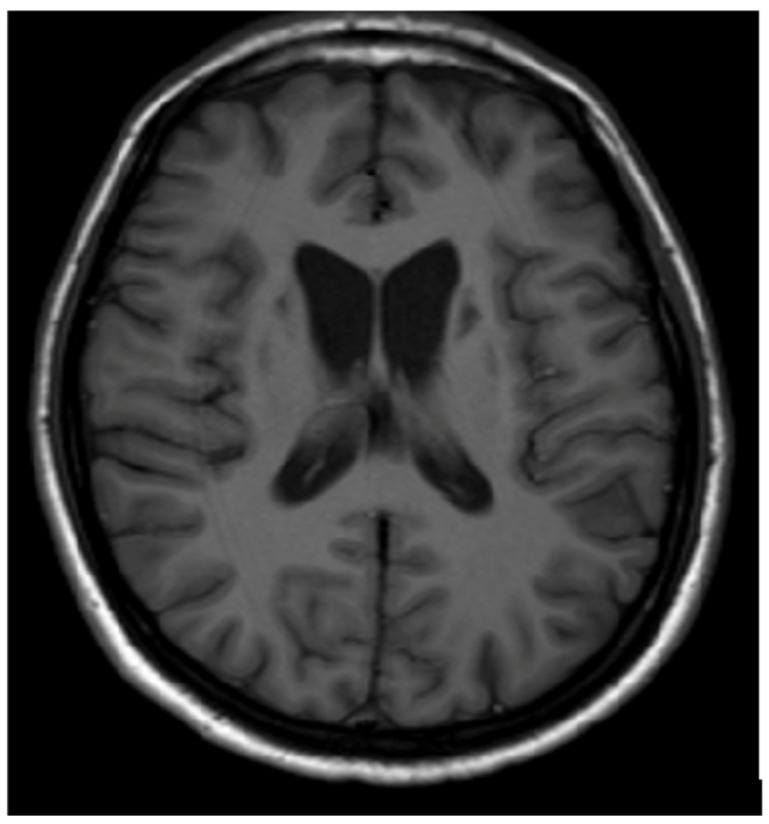
Hypointense changes localized in both putamen in T1-weighted images (own materials of the neurology department).

In cases of advanced disease (long-term and untreated), brain MRI changes may appear as hypointense areas on T1-weighted images (necrosis or atrophic changes) [12,13,14,15,16,17,18,19,20,21] (Figure 3). It should be noted that several metabolic, genetic, neoplastic, and inflammatory disorders can involve the basal ganglia symmetrically. Occasionally, these disorders also affect other brain structures, sometimes unilaterally. Brain MRI plays a supportive role in diagnosis and aids in differential diagnosis; however, without clinical symptoms and additional clinical, metabolic, genetic, etc., examinations, brain MRI alone cannot form the basis for disease diagnosis [1,2,3].

**Figure 4:**
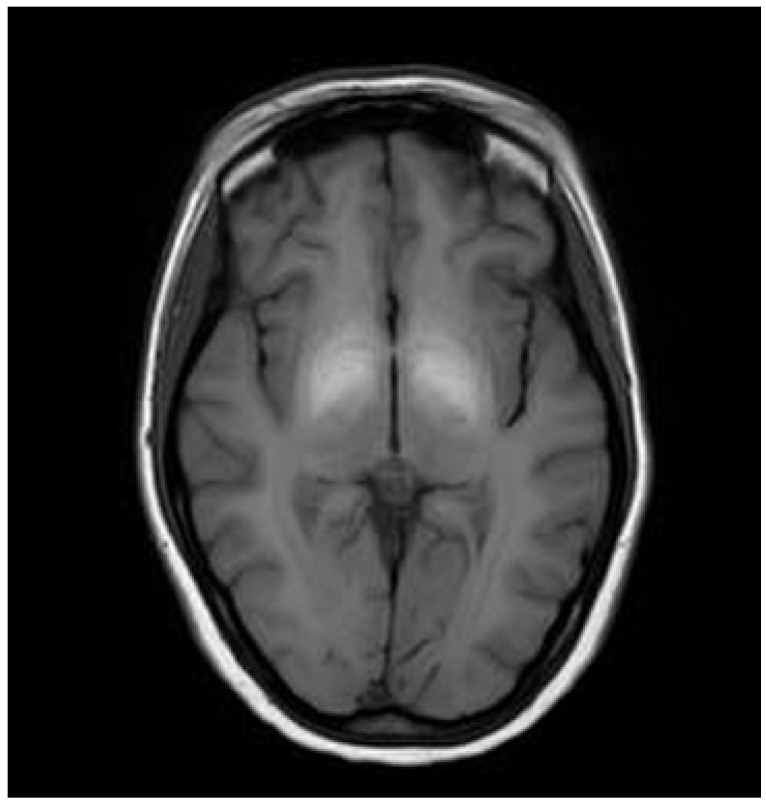
Hyperintense changes localized in both globii pallidi in T1-weighted images (probably due to manganese accumulation) (own materials of the neurology department).

In some cases, white matter (up to 20% of WD patients) and the corpus callosum may also be affected. As WD is primarily a hepatic disorder, severe liver pathology (liver failure, hepatic encephalopathy (HE)) can result in symmetrical hyperintense changes in the globus pallidus and substantia nigra on T1-weighted images, likely due to manganese intoxication similar to those observed in acquired hepatocerebral degeneration (AHD) [20] (Figure 4). However, these changes are not typical of WD but are more characteristic of AHD or HE.

The typical brain MRI changes described above occur in nearly 100% of WD patients with neurological symptoms, 42–70% of those with the hepatic phenotype, and even in 20% of presymptomatic cases [22]. These data contributed to the inclusion of brain MRI in the Leipzig score since 2003, which is part of the basic diagnostic algorithm for WD [23]. In this scoring system, which consists of clinical (Kayser–Fleischer ring, presence of neuropsychiatric symptoms, brain MRI, hemolytic anemia) and laboratory findings (copper metabolism, genetics), the characteristic brain MRI findings for WD are scored as two points. A diagnosis of WD is established when the total score is four points or more. Another benefit of brain MRI in WD is its utility for disease monitoring, with several studies documenting the resolution of WD-related brain MRI changes during treatment [1,3,24,25,26,27,28], as well as white matter changes due to overtreatment (copper deficiency) [25,26,27]. Additionally, data suggest that brain MRI lesions located in the pons or thalami may be more frequently associated with early neurological deterioration during WD treatment [24]. In summary, classical brain MRI in WD is a well-established procedure recommended by hepatologists and neurologists for both diagnosis and monitoring of the disease.

However, physicians should be aware that bilateral lesions of the basal ganglia and thalami may occur in several other disorders, including metabolic conditions (glutaric aciduria type I, methanol intoxication, hepatic encephalopathy, uremic encephalopathy, hypoglycemia, hyperglycemia, carbon monoxide poisoning, toluene toxicity, acute hyperammonemic encephalopathy), mitochondrial diseases (Leigh syndrome), genetic disorders (neurodegeneration with brain iron accumulation (NBIA), Huntington disease, primary familial brain calcifications, gangliosidosis GM1 and GM2), autoimmune encephalitis, cryptococcosis, and many others [29]. The results of brain MRI may only suggest and support the diagnosis, but the course of the disease, age of onset, additional laboratory results, and genetic studies are key to making a correct diagnosis [29].

### 3.2. Diffusion MRI in WD

Magnetic resonance diffusion imaging enables the assessment of tissue microstructure by quantifying the random movement (Brownian motion) of water molecules [30,31]. The biophysical parameter that quantifies the speed and extent of movement of water molecules irrespective of their direction is the apparent diffusion coefficient (ADC) [30,31]. ADC maps depict the distribution of diffusion within examined tissues, averaging over the population of water molecules in each voxel [30]. The orientation of diffusion is assessed through diffusion tensor imaging (DTI), which provides data on three primary parameters: mean diffusivity (MD), fractional anisotropy (FA), and the principal directions of diffusion—radial diffusivity (RD) and axial diffusivity (AD) [30,31]. MD describes the average displacement of molecules and the general impediments to diffusion within tissues [30,31]. FA quantifies the variability of particle movements in space and correlates with tissue structure coherence [30,31]. The principal direction of diffusivity is indicative of the spatial alignment of tissue structures [30,31].

**Figure 5:**
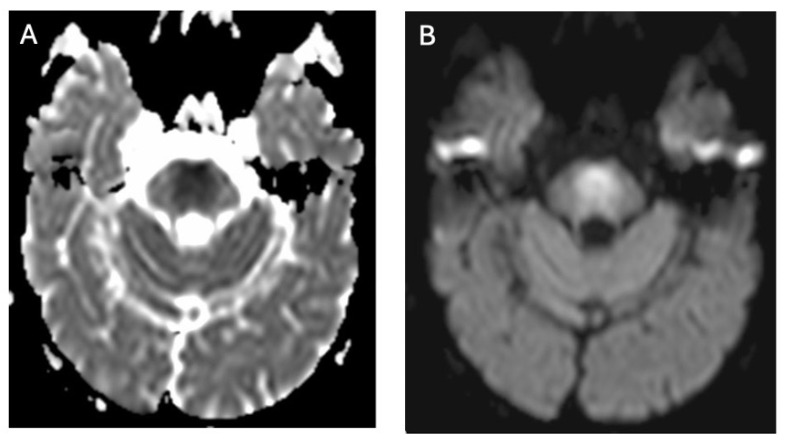
Neuroimaging findings of WD affecting the pons—a hypointense signal on ADC maps (**A**) and a hyperintense signal on diffusion-weighted imaging (DWI) (**B**), indicative of cytotoxic edema (own materials of the neurology department).

In WD, diffusion abnormalities can manifest as both restricted and increased diffusion (Figure 5). Diffusion restriction is relatively uncommon and typically appears in the early stages of the disease, presenting as hyperintense foci on T2-weighted and FLAIR images, indicative of edematous changes [32,33,34,35,36,37]. Increased diffusion, observed in structures such as the putamen, globus pallidus, internal capsules, midbrain, pons, and white matter, likely reflects neuronal loss, gliosis, and spongiosis [36].

### 3.3. Diffusion Tensor Imaging (DTI)

DTI studies in the white matter revealed elevated MD and reduced FA. These changes were observed in the basal ganglia, thalamus, internal capsules, corpus callosum, corona radiata, and the white matter of the frontal and occipital lobes [33,38,39]. White matter lesions appeared in both signal-altered and normal-appearing regions on conventional MRI images [33], underscoring DTI’s capability to detect early lesions that are otherwise imperceptible on standard MRI scans.

### 3.4. Brain Iron Accumulation in WD (SWI, T2*, and QSM)

According to the pathogenesis of WD, copper accumulation in various organs and tissues leads to clinical symptoms [40]. However, several studies have also documented iron accumulation in the liver and brain of WD patients [40,41,42,43,44]. This phenomenon is attributed to the diminished ferroxidase activity of Cp, which disrupts iron transport (required for its incorporation into transferrin and circulation) and triggers inflammatory reactions in the liver and brain due to copper buildup, leading to phagocytic cell infiltration and secondary iron deposition. Initial investigations suggesting brain iron accumulation in WD were based on T2-weighted brain MRI sequences, where hypointense signals were interpreted as potential iron accumulation [45,46,47]. Subsequent studies utilizing advanced MRI techniques such as susceptibility-weighted imaging (SWI), gradient echo (T2*), relaxometry (R2*), and QSM provided indirect evidence supporting brain iron accumulation in WD, particularly in the basal ganglia and among patients with neurological symptoms [48,49,50,51,52,53]. Post-mortem studies using 7T MRI (nine patients with WD; seven neurologic, two hepatic phenotype, and six controls) further corroborated these findings, confirming in vivo that the hypointensity observed in T2 sequences reflects iron accumulation, predominantly attributable to neurodegeneration and the influx of phagocytic cells [48]. The significance of brain iron accumulation in WD has led to the inclusion of SWI/T2* sequences documenting such changes in the brain MRI semiquantitative scale proposed by Dusek et al. [53].

### 3.5. Volumetric Studies in WD

**Figure 6:**
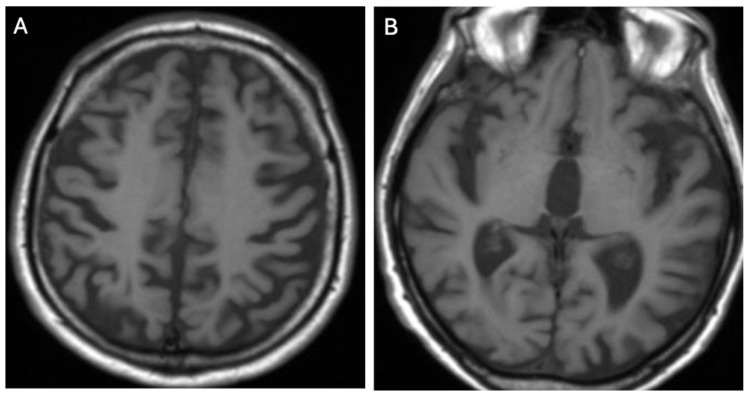
Cortical atrophy (**A**) and central atrophy with widening of the third ventricle (**B**) in T1-weighted sequences (own materials of the neurology department).

In the course of Wilson’s disease (WD), alongside lesions predominantly located in the basal ganglia, mesencephalon, and pons, anatomopathological studies have consistently described the WD brain as soft, exhibiting loss of both deep and superficial white matter, slight atrophy, and enlarged ventricles [54,55,56]. Since the advent of neuroimaging studies in WD (using brain CT or MRI), brain atrophy has been documented with cortical or ventricular widening observed in nearly 40% of patients, particularly those with neurological manifestations (204 patients with WD) [22]. Initially, radiologists subjectively assessed atrophy (presence/absence) [49,56,57,58,59,60] (Figure 6A,B).

In subsequent studies, traditional linear brain measurement indices, such as the Huckman number, are defined as the sum of the maximum distance between the anterior horns and the minimum distance between the bicaudate nuclei. This parameter is useful for evaluating ventricular enlargement, particularly the diameter of the anterior ventricular horn, third ventricle width, ventricular index, and sulcus width, which were utilized to quantify brain atrophy, particularly in patients with neurological manifestations of WD [57,58]. As the analysis of brain atrophy evolved with the introduction of objective software tools similar to those used in multiple sclerosis (which are even used as secondary endpoints in clinical trials), studies began employing software like SIENAX, FreeSurfer (2.6/FSL 6.0), (and voxel-based morphometry to assess brain parenchyma in WD. One of the pioneering studies utilizing SIENAX was conducted by Smolinski et al., who examined 48 treatment-naïve WD patients and observed correlations between neurological deficits, functional impairment (as measured by Unified Wilson’s Disease Rating Score (UWDRS)—clinical scale for patients with WD describing the severity of neurological symptoms and ambulation), and total brain volume, as well as volumes of white and gray matter [59]. The authors further noted associations between ‘toxic copper’ (NCC) levels and reduced brain volumes. In a subsequent longitudinal investigation, the authors analyzed baseline and follow-up brain MRIs of WD patients over a period exceeding 12 months (fifty-seven patients with WD; thirty-six neurological, seventeen hepatic, and four presymptomatic). They found that the annualized rate of brain atrophy, defined as the longitudinal percentage change in ventricular volume (PVVC), was notably higher in neurological WD patients (median 5.4%) compared to non-neurological WD patients (0.5%, similar to healthy populations). Furthermore, the extent of atrophy varied depending on the neurological subtype (e.g., more severe forms like dystonia exhibited 14%, parkinsonian 7.9%, and tremor 4.3%) and disease progression—patients experiencing neurological deterioration showed rates as high as 16.7% [56].

More comprehensive whole-brain analyses employing deformation and surface-based morphometry were conducted by Dusek et al. in 2021 [49] (29 patients with WD and 26 controls), revealing atrophy affecting deep gray matter nuclei, brainstem, internal capsule, motor cortex, and corticospinal cortex in WD patients. These findings were corroborated by voxel-based morphometry and region-of-interest volumetric analyses performed by Shribman et al. [55], who also observed reduced gray matter volumes in basal ganglia, thalamus, brainstem, cerebellum, anterior insula, and orbitofrontal cortex among neurological WD patients compared to hepatic WD patients (40 patients with WD, 23 neurological, and 17 hepatic). The severity of neurological deficits, as previously noted, correlated with the extent of neurological symptoms assessed using UWDRS.

The findings from these studies, which underscore brain atrophy resulting from copper toxicity in WD [59,60,61,62,63,64,65], strongly advocate for the use of longitudinal volumetric studies as an objective biomarker of neurological disease in WD in future research and in the clinical management of WD patients.

## 4. Brain MRI Scales in WD

Due to the clinical symptoms’ heterogeneity in WD, management and treatment monitoring, similar to other disorders, require validation and control using objective scales. Based on the pathogenesis of WD, treatment monitoring typically relies on copper metabolism parameters such as serum copper levels, NCC, direct NCC, and daily urinary copper excretion [1,2,3]. These parameters are crucial for assessing compliance with anti-copper treatment and monitoring copper metabolism to prevent copper deficiency. Given the multiorgan involvement in WD, additional evaluation with clinical and laboratory scales is warranted, particularly to assess liver function and neurological deficits. For hepatic WD assessment, severity can be evaluated using the Model of End Stage Liver Disease (MELD), Nazer Score, New Wilson Index for liver failure, and fibrosis indices such as the Fibrosis-4 (FIB-4) Index for Liver Fibrosis [6].

The first three scores are based on laboratory results and are primarily used to prioritize liver transplant allocation and predict patient survival before transplantation. The serum parameters necessary for calculating the MELD score include serum sodium level, bilirubin, creatinine, and the international normalized ratio for prothrombin time (INR). The Nazer Score and the New Wilson Index scales are also used for liver transplant qualification, particularly in WD patients, but they utilize serum INR, bilirubin, aspartate aminotransferase levels, and in the case of the New Wilson Index, additionally consider serum white blood cell count and albumin levels. The FIB-4 index is a non-invasive method based on patient age, serum levels of alanine and aspartate aminotransferases, and blood platelet count, which helps identify the stage of liver fibrosis [1,2].

In neurological assessment, clinical scales such as the Unified Wilson’s Disease Rating Scale (UWDRS) or the Global Assessment Scale for Wilson’s Disease (GAS for WD) are utilized. The UWDRS, designed for WD patients, comprises three parts: (1) consciousness assessment, (2) activities of daily living (ADL), and (3) detailed neurological examination. The GAS for WD is structured into two tiers: Tier 1 assesses global disability across four domains (liver, cognition/behavior, motor, and osseomuscular), while Tier 2 focuses on a detailed neurological examination. Recognized by the Movement Disorders Society (MDS), the GAS for WD serves as a standard scale in clinical trials and is utilized in European and North American registries. Unlike the UWDRS, which does not include liver symptoms, cognition, and psychiatric symptoms (all crucial in WD), the GAS for WD provides a detailed assessment of neurological examinations, ADL, and ambulation of WD patients [1,2]. Country-specific osseomuscular deformities are covered in the GAS for WD, although they are less frequently observed in other global regions. Nevertheless, both scales are validated for WD and widely employed to objectively document clinical severity [1,2].

However, since the introduction of brain MRI, several studies have sought objective neuroradiological scales to assess the severity of brain injury in WD, as described below [1,2,3,4].

### Brain MRI Scoring Systems in WD

Scoring systems play a crucial role in standardizing the assessment of imaging tests. One of the pioneering attempts to assess the severity of neuroradiological manifestations in Wilson’s disease (WD), conducted by Prayer et al., utilized brain MRI to evaluate criteria such as enlargement of internal/external liquor spaces, focal lesions, basal ganglia lesions, white matter lesions, and brain stem lesions (38 patients with WD and 40 controls). This study aimed to correlate these findings with the severity of neurological symptoms, which were graded on a scale from 0 to 3 [13]. Another classification proposed by Kim et al. categorized 50 WD patients based on brain MRI signal abnormalities into three distinct groups: (1) those with normal brain MRI findings; (2) those exhibiting abnormal high signal intensity on T1-weighted sequences (potentially indicative of hepatic encephalopathy or manganese accumulation); and (3) those showing abnormal high signal intensity on T2-weighted images [46]. Notably, the authors observed significant improvement in neuroradiological findings among patients in group 3 (six out of nine cases, representing 67%) following anti-copper therapy. The first quantitative brain MRI study utilizing T1, T2, and FLAIR sequences in WD was conducted by Sinha et al. in 2006 (100 WD patients) [15]. The study comprehensively analyzed various brain regions, including the caudate nucleus, putamen, internal capsule, thalamus, midbrain, pons, medulla, cerebellum, white matter, and cortex. Sinha et al. proposed a signal grading system: 0 for no abnormality, 1 for signal intensity changes without atrophy, 2 for signal intensity changes with mild to moderate atrophy, and 3 for signal intensity changes with severe atrophy. The cumulative score, termed MRI severity index, was shown to correlate with neurological symptoms scores (NSS). Another quantitative brain MRI study by da Costa et al. focused on evaluating affected structures, including the putamen, caudate nucleus, thalamus, pons tegmentum, globus pallidus, midbrain tegmentum, middle cerebellar peduncle, periaqueductal gray matter, centrum semiovale, and substantia nigra, using T2 and Proton density (PD) weighted sequences in 18 WD patients [27]. The severity of brain MRI findings was quantified by assigning points, with a maximum score of 10 points indicating the involvement of all structures. Poujois et al. proposed (48 drug naïve patients with WD) another brain MRI scoring system, initially adopted by their French group, which assessed abnormal signals observed in T2-weighted, FLAIR, and T1 sequences in specific brain regions, including the lenticular nucleus, caudate nucleus, thalamus, mesencephalon, pons, and dentate nucleus (scores ranged from 0 to 6 according to location) [66]. They found a positive correlation between their neuroradiological scale and exchangeable copper levels (CuEXC). A similar neuroradiological scale was proposed by the Polish group led by Litwin et al. in 2020, based on lesion counts detected in T2 and FLAIR weighted sequences across various brain structures, such as the putamen, globus pallidus, caudate nucleus, thalamus, mesencephalon, pons, substantia nigra, cerebellum, and measures of brain atrophy (100 drug naïve patients with WD) [67]. The number of lesions identified correlated with the UWDRS, though, like previous scales, it did not account for the potential reversibility of changes or incorporate newer sequences like SWI, T2*, or quantitative brain atrophy assessments.

Currently, the most compelling scale for analyzing brain MRI in WD (39 patients with WD) is the semiquantitative scale proposed by Dusek et al. in 2020 (Table 1) [53]. This scale innovatively incorporates new sequences, such as SWI, T2*, and volumetry, aiming to distinguish reversible and irreversible lesions according to WD pathogenesis. The scale is structured into acute toxicity score, chronic damage score, and total score, evaluated across brain structures typically affected in WD, including the putamen, caudate nucleus, globus pallidus, thalamus, mesencephalon, pons, and nucleus dentatus. Detailed assessments of brain atrophy (cortical, central, midbrain, and cerebellar) are also integral to the scale. The acute toxicity score, based on hyperintense signals in T2/FLAIR sequences within these structures, reflects edema and demyelination—processes potentially reversible in nature. In contrast, the chronic damage score, characterized by hypointense signals in T2/T2*/SWI sequences, primarily signifies necrosis and iron accumulation due to macrophage influx into necrotic brain lesions, often accompanied by irreversible brain atrophy on MRI. The initial pilot study introducing this scale [53], subsequent validation studies [68] (100 patients with WD), and the inclusion of additional biomarkers like neurofilament light chains (sNfLs) (61 drug naïve patients with WD) [69] have underscored the reliability of this approach. Brain MRI scores from this semiquantitative scale correlate significantly with the severity of neurological disease as assessed by UWDRS, as well as with sNfL levels, indicative of neuronal injury. Higher scores in the brain MRI semiquantitative scale at baseline predict a greater likelihood of neurological deterioration in WD, particularly reflecting chronic damage, which may delineate the natural progression of the disease. This scale holds promise for enhancing brain MRI analysis in WD; however, efforts are underway to develop software that can automate scoring without requiring direct physician input.

Finally, building upon this scale and data from diffusion-weighted sequence analyses in WD, a research group from China proposed a cranial diffusion-weighted imaging scale for WD in 2023 [70]. In addition to T1-, T2-, and FLAIR-weighted sequences, they incorporated DWI sequences, highlighting hyperintensity on DWI as indicative of acute damage, thereby enhancing the scale’s diagnostic value. Lesions in WD were evaluated across brain regions, including the putamen, globus pallidus, head of caudate nucleus, internal capsule, thalamus, midbrain, pons, medulla oblongata, cerebellum, cortex, and corpus callosum, with scoring criteria of 0/1 for T1, T2, and FLAIR sequences, brain atrophy on T1 (0/1), and 0–2 for DWI. This scale, based on 123 patients with WD, demonstrated positive correlations with UWDRS parts II and III, as well as with the brain MRI semiquantitative scale. Notably, detailed analyses indicated that DWI hyperintensity in the putamen might serve as a prognostic indicator for neurological deterioration. Moreover, each incremental increase in this neuroimaging score was associated with a 5.2% higher risk of neurological decline. However, further validation of this scale across multiple centers and by diverse research groups remains necessary.

In summary, among the neuroradiological clinical scales used in WD, the semiquantitative brain MRI scale shows significant promise as the principal tool for clinicians managing WD patients. It has been employed in several studies from different countries. We have provided more detailed descriptions of other scales only as proposals, with Table 1 presenting the brain semiquantitative scale proposed by Dusek [53]. However, for enhanced objectivity in application, the development of artificial intelligence software capable of analyzing brain MRI images according to the scale’s criteria is essential.

## 5. Neuroradiological Pathognomonic Signs of WD

In addition to typical and frequently encountered features among patients with WD, especially in its neurological form, hyper- and hypointense changes in deep brain structures in T2, T2*, FLAIR, and SWI sequences on brain MRI have been noted [10]. Hyperintense foci in the midbrain and pons may sometimes occur in a characteristic pattern with normal brain tissue. Characteristic signs include the ‘face of the giant panda’ in the midbrain and the ‘miniature panda’ sign in the pons, which are considered so-called pathognomonic neuroradiological signs of WD. Besides the aforementioned abnormalities, other pathognomonic signs in WD include the bright claustrum sign, split thalamus (onion sign), and whorl sign [71].

**Figure 7:**
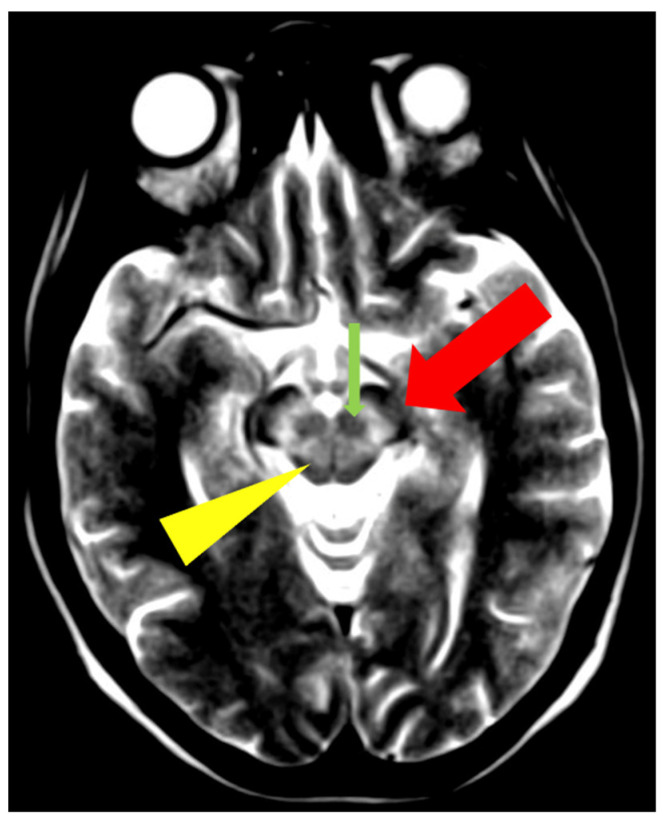
The ‘face of the giant panda’ sign—increased signal intensity in the midbrain tegmentum and hypointense red nuclei (thin arrow), the substantia nigra (thick arrow), and hypointense signal in the superior colliculi (arrowhead) (own materials of the neurology department).

**Figure 8:**
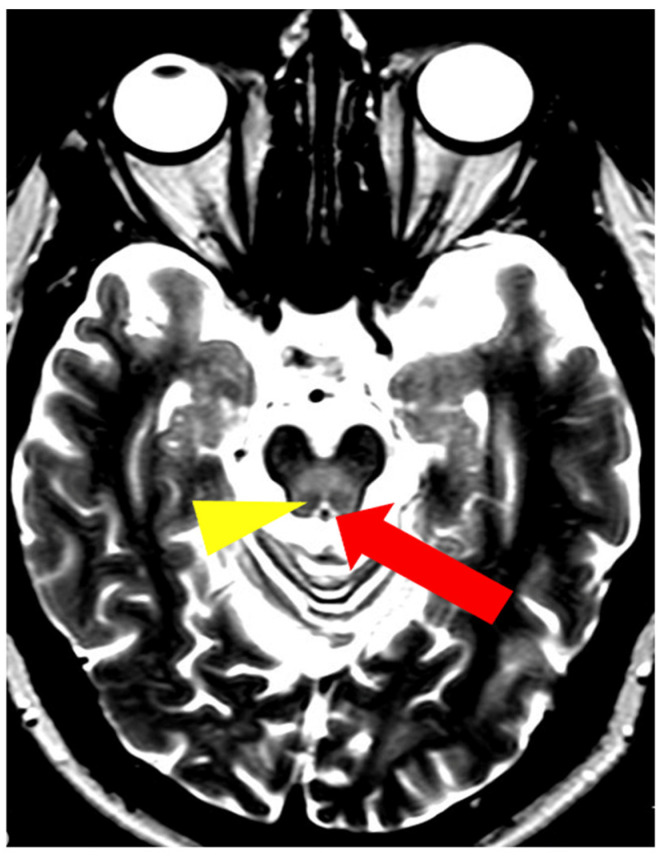
The ‘miniature panda’ sign—decreased signal intensity in the medial longitudinal fasciculi and tegmental tracts (arrowhead), hyperintensity of the aqueduct opening into the fourth ventricle (arrow) (own materials of the neurology department).

**Figure 9:**
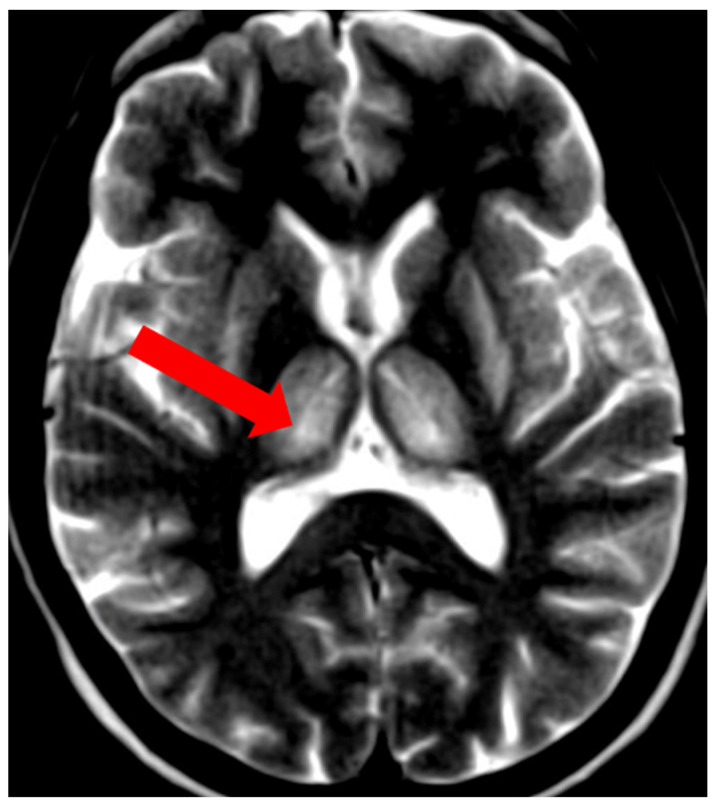
The split thalamus sign—increased signal intensity of the internal medullary lamina between the medial and lateral groups of thalamic nuclei (arrow) (own materials of the neurology department).

**Figure 10:**
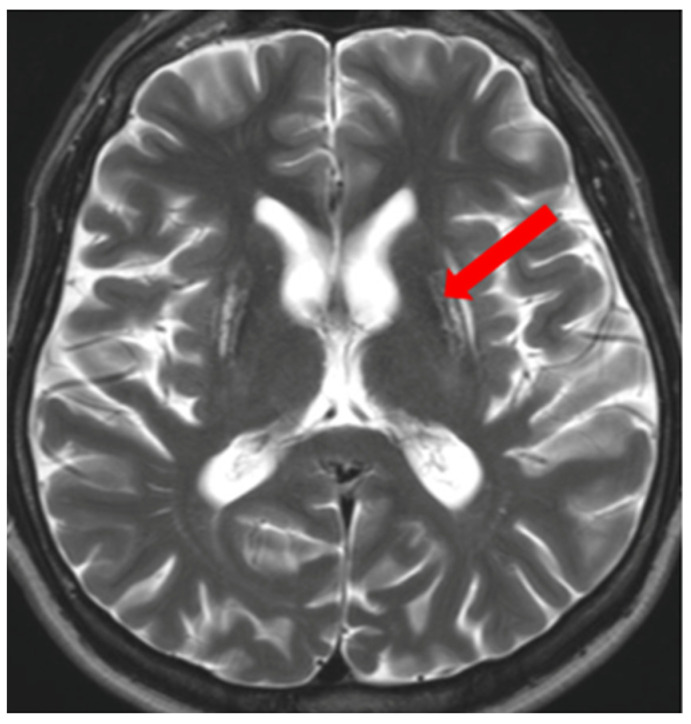
The whorl sign—hyperintense bands in the putamen (arrow) (own materials of the neurology department).

**Figure 11:**
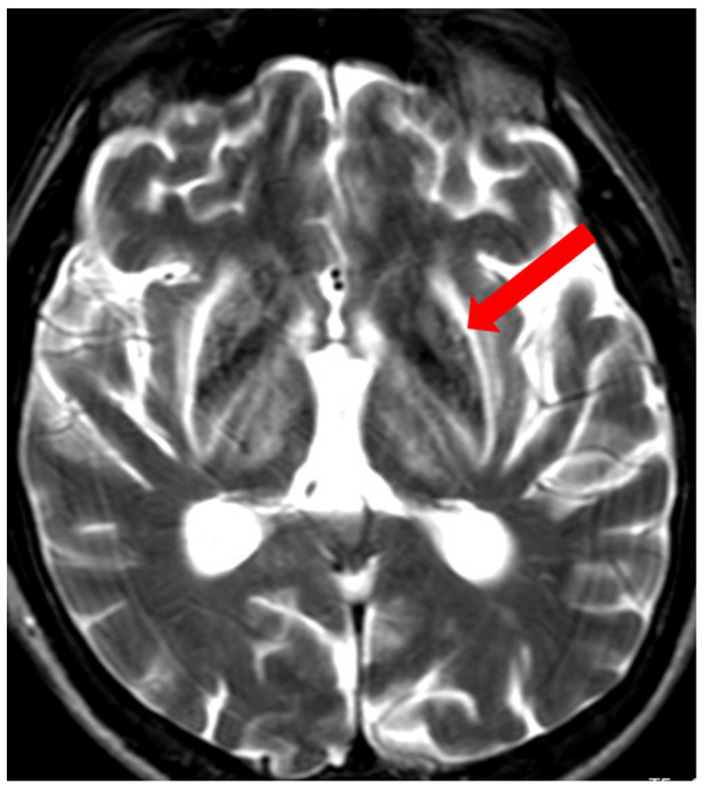
The bright claustrum sign—increased signal intensity in the claustrum (arrow) (own materials of the neurology department).

The ‘face of the giant panda’ sign is characterized by an area of increased signal intensity in the midbrain tegmentum and hypointense red nuclei (panda’s eyes), the substantia nigra forming a reticular structure (panda’s ears), and hypointense signal in the superior colliculi (panda’s chin) (Figure 7) [71,72]. The ‘miniature panda’ sign is observed in the pontine tegmentum and consists of hypointense medial longitudinal fasciculi and tegmental tracts (panda’s eyes), hyperintensity of the aqueduct opening into the fourth ventricle (panda’s nose and mouth), with the superior cerebellar peduncles forming the panda’s cheeks (Figure 8) [71,72]. It may appear independently or in conjunction with the ‘face of the giant panda’ sign. In T2 and FLAIR sequences, the presence of a hyperintense internal medullary lamina between the medial and lateral groups of thalamic nuclei has been noted—the split thalamus sign arises from the internal medullary lamina system with significantly increased signal intensity separating the medial and lateral thalamic nuclei with higher signal intensity but lower than the internal lamina (Figure 9) [73]. The whorl sign arises in the putamen due to the coexistence of several concentrically arranged hyperintense bands [15] (Figure 10). The bright claustrum sign arises from the increased signal intensity in the claustrum [74] (Figure 11). In the publication by Su et al., another neuroradiological sign was presented using 7T SWI brain MRI. The hyperintense globus pallidus rim sign was defined as a linear pseudohyperintense signal at the lateral border of the globus pallidus, resulting from the hypointense signal of the globus pallidus and putamen [75].

In the study by Rędzia-Ogrodnik et al. [71], among patients with neurological symptoms of WD (n = 55), the most frequently occurring pathognomonic neuroradiological sign, present in 27.3% of cases (15/55), was the ‘face of the giant panda’ sign, followed by the ‘miniature panda’ sign (21.8%, 12/55 neurological patients), and the split thalamus sign (12.7%, 7/55 neurological patients), while the whorl and bright claustrum signs occurred in only one patient (1.8%, 1/55) [70]. In another study, Prashanth et al. identified the ‘face of the giant panda’ sign in 14.2% (8/56) of WD patients with neurological symptoms [76].

In conclusion, neuroradiological signs considered as pathognomonic are present among patients with the neurological form of WD and may contribute to expediting diagnosis. They may not be truly pathognomonic as most of these signs have also been described in other neurological disorders (e.g., the ‘face of the giant panda’ sign in Leigh syndrome or the split thalamus sign in fucosidosis type 1) [51,73,74,75,76,77,78].

## 6. Discussion

Currently, following international recommendations, brain MRI utilizing classical sequences (T1, T2, FLAIR) is essential for every patient undergoing evaluation for WD, forming a crucial component of the diagnostic protocol (Leipzig score) [1,2]. Understanding the so-called ‘pathognomonic neuroradiological signs of WD’ aids in distinguishing WD from other extrapyramidal disorders, although it is noteworthy that these signs may rarely manifest in other conditions [68]. However, it is important to note that several other disorders (neurological, metabolic, etc. [29]) can present brain MRI findings similar to those seen in WD, potentially leading to misdiagnosis of WD and delaying the diagnosis of the underlying disease. The Leipzig scoring system awards two points for typical symmetrical brain MRI lesions or pontine lesions in suspected WD patients, which can be both helpful and pose a risk of overinterpretation in WD diagnosis [1,2,23]. Physicians should bear in mind that diagnosing WD is complex and should rely on disturbances in copper metabolism, clinical symptoms (including hepatic and/or neuropsychiatric symptoms that may manifest as brain MRI changes), and genetic testing. A definitive diagnosis of WD should be based on consistent findings from these examinations [1,2,23].

Prospective observations from longitudinal studies in WD have documented that, during anti-copper therapy or after LT, brain MRI changes in some WD patients diminish or even resolve [1,2]. Kim et al. analyzed nine WD patients with hyperintense signals in the basal ganglia on T2-weighted sequences and found improvement in six patients (67%), stable images in two patients (22%), and worsening in one patient (11%) [46]. Magalhaes et al. also demonstrated that WD patients with a short delay in anti-copper treatment (1–3 years) showed clinical and neuroradiological improvement (improvement in four out of five patients; 80%) [26]. Additionally, case reports of patients post-LT support the possibility of reversal of brain MRI pathology [79]. A rare clinical problem that can occur in treated WD patients, sometimes detectable on brain MRI, is ‘overtreatment’, which can cause white matter changes and epilepsy [80]. Lastly, the brain MRI semiquantitative scale proposed by Dusek et al. documented the reversibility of changes (total score as well as acute toxicity score), emphasizing the importance of periodic brain MRI monitoring in WD patients [53].

Given all these data, as well as the potential reversibility of brain MRI abnormalities with anti-copper treatment, regular imaging during follow-up visits is recommended, particularly for patients experiencing neurological decline (due to copper deficiency during treatment or liver dysfunction leading to brain manganese accumulation).

Furthermore, data on lesion localizations (such as in the thalamus and pons) suggest a correlation with the potential for early neurological deterioration in WD patients at the initiation stage of anti-copper treatment [24]. These findings imply the need for cautious initiation of anti-copper therapy, including titration of chelators or even preference for zinc salts in treatment protocols [24]. All these findings underscore the importance of classical brain MRI neuroimaging in the differential diagnosis, diagnosis, and treatment of WD, as outlined in international recommendations for WD treatment. Currently, promising neuroradiological scales proposed for brain MRI in WD hold the potential for enhancing disease monitoring; however, they need to be further investigated (different scales, as well as performed on scanners up to 1.5 T—not verified on a scanner with higher magnetic fields) [48,49]. However, enhancing the objectivity of scoring could benefit from the implementation of artificial intelligence (AI) software; unfortunately, such tools are currently unavailable for use.

Brain volumetric studies, integral to the brain MRI semiquantitative scale in WD, have yielded intriguing insights into copper toxicity-related brain atrophy, primarily affecting grey matter. However, it should be emphasized that most studies analyzing brain atrophy in WD are single studies conducted on a limited number of patients. These studies often use different software (e.g., Freesurfer, SIENAX) or rely on subjective visual assessments performed by physicians, such as in the case of the brain semiquantitative scale [57,58,59,60]. Future longitudinal studies should further explore brain volumetrics as an endpoint biomarker for neurodegeneration in WD, especially using AI (independent for physicians’ tools). While new brain MRI sequences such as DWI, SWI, and advanced postprocessing techniques like QSM show promising results, their use remains predominantly in research rather than routine clinical management of WD. Different MRI scanners and magnetic fields (from 0.3 T to 7 T), especially in these sequences, give completely different results. Patients with nearly normal basal ganglia on a 0.3 T scanner may show images suggesting iron accumulation on a 3 T (or higher field) scanner [48,49]. The lack of standardization of MRI scanners remains a significant obstacle to the advancement of new MRI techniques in WD until AI can provide a solution. Looking ahead, researchers should explore techniques such as free water diffusion, MRI using higher magnetic fields like 7 T (however, limited due to high costs, as well as due to low availability and possible medical and non-medical contraindications) [75], and specialized MRI methods that focus on metals and cell types (targeting copper, iron, manganese, neurons, and astrocytes). These advancements have the potential to significantly improve WD monitoring capabilities.

All these studies had significant limitations. Primarily, they involved a limited number of patients (typically up to 30–40) who were in various stages of the disease. However, it is important to note that WD is a rare condition, making it very challenging to gather a large cohort of drug-naïve WD patients at a single center using the same MRI device.

Another significant source of potential bias stems from the specialization of the centers involved. Hepatic centers primarily treat patients with hepatic symptoms, whereas neurological centers tend to have patients with neurological manifestations. This specialization can impact the study results. Additionally, as previously mentioned, the use of different brain MRI devices may significantly affect the results, particularly with newer sequences like SWI, T2*, and QSM [81,82,83,84,85,86]. SWI is routinely used in clinical MRI and, compared to T2*, demonstrates improved sensitivity in detecting iron in the brain. However, there are intrinsic disadvantages to SWI. Firstly, air tissue artifacts in SWI interfere with the assessment of brain regions adjacent to the temporal bone and sinuses. Secondly, blooming artifacts may sometimes lead to errors in normal tissue signals and the loss of anatomical borders [81]. Thirdly, the coexistence of iron and calcium deposits may result in confusing signal intensity patterns [82]. Additionally, SWI, like T2*, allows only qualitative assessment of brain iron content without quantitative evaluation. QMC is a new, sophisticated post-processing technique representing the quantitative extension of SWI [83]. It eliminates the blooming artifacts seen in SWI [84] and can distinguish between paramagnetic substances such as iron and diamagnetic substances such as calcium [85]. Although QSM reconstruction algorithms are rapidly developing and optimizing [86], the process is still time-consuming and, therefore, impractical for routine clinical applications.

## 7. Conclusions

Based on our review and current international recommendations from the European Association for the Study of the Liver (EASL) [1] and the American Association for the Study of Liver Diseases (AASLD) [2], a brain MRI examination is recommended before initiating treatment in all patients with neurological WD. It should be included as part of the evaluation for any patient presenting with neurological symptoms to establish a baseline status and to exclude other potential causes.

Based on recommendations for neurological follow-up in these patients, brain MRI should also be considered as part of long-term treatment monitoring during neurological examinations. New MRI sequences and brain MRI scales, including the semiquantitative scale proposed by Dusek et al. [53], show promise. However, they are currently in the research stage and require further investigation, especially multicenter studies.

## Figures and Tables

**Table 1:** Brain MRI WD semiquantitative scale—scoring system (proposed by Dusek et al., 2020) [53].

	Normal/Absent	Mild/Moderate	Severe
Acute toxicity score (evaluated as hyperintensity on T2-weighted and FLAIR sequences)			
Putamen	0	1	2
Caudate nucleus	0	1	2
Thalamus	0	1	2
Mesencephalon	0	1	2
Pons	0	1	2
Other areas (sepcify)	0	1	2
Chronic damage score (evaluated as hypointensity on T2/T2*/SWI sequences)			
Globus pallidus	0	1	
Putamen	0	1	
Caudate nucleus	0	1	
Thalamus	0	1	
Dentate nucleus	0	1	
Atrophy (assessed on T1 sequences)			
Cortical	0	1	2
Central	0	1	2
Midbrain	0	1	2
Cerebellar	0	1	2

## Data Availability

No new data were created or analyzed in this study. Data sharing is not applicable to this article.
